# Factors Associated with Successful Smoking Cessation According to Age Group: Findings of an 11-Year Korea National Survey

**DOI:** 10.3390/ijerph18041576

**Published:** 2021-02-07

**Authors:** Youngmee Kim, Ji Sung Lee, Won-Kyung Cho

**Affiliations:** 1 Red Cross College of Nursing, Chung-Ang University, Seoul 06974, Korea; youngkim234@gmail.com or; 2 College of Medicine, University of Ulsan, Seoul 05505, Korea; totoro96a@gmail.com; 3 Asan Institute for Life Sciences, Clinical Research Center, Asan Medical Center, Seoul 05505, Korea; 4 Department of Pulmonary and Critical Care Medicine, International Healthcare Center, Asan Medical Center, Seoul 05505, Korea

**Keywords:** age, male, smoking, cessation, Koreans

## Abstract

Previous studies suggest that factors related to smoking cessation may vary with age. This study examined the factors affecting smoking cessation by age among Korean adult male smokers using data from the Korea National Health and Nutrition Examination Survey from 2007 to 2018 (excluding 2013). Logistic regression analyses were used to investigate various factors associated with smoking cessation in three different age groups. Out of a total of 15,492 individuals, 31.5% of the 3523 individuals aged 19–39 years (young adult), 54.7% of the 7390 individuals aged 40–64 years (middle-aged), and 78.6% of the 4589 individuals aged ≥65 years (older adults) succeeded in quitting. In the young adult and middle-aged groups, being married was associated with successful quitting, and lifetime smoking was associated with unsuccessful quitting. Willpower and several comorbidities were related to successful cessation in the middle-aged and older adult groups. Skipping any meal, which suggests unhealthy behavior, was negatively related to quitting in the young adult group. We observed that factors associated with smoking cessation success or failure differed by age, which should be considered when developing smoking cessation policies and programs.

## 1. Introduction

Although the number of smokers is decreasing worldwide, more than 1.1 billion people reportedly smoked in 2015 [1]. Smoking remains a critical global public health issue. Most smokers who are aware of tobacco’s harm want to quit but they often fail. Numerous physiological, behavioral, environmental, psychological, cognitive, and social factors are associated with quitting success or failure [2,3,4,5,6].

In South Korea, the smoking rate for adult males was reported to be 38.1% and 36.7% in 2017 and 2018, respectively [7]. A national survey that investigated the factors influencing successful smoking cessation in adult Korean males found age to be a key factor. Specifically, the success rate of quitting smoking was significantly higher in older men [8]. However, evidence regarding the relationship between age and smoking cessation remains inconsistent. A few previous studies, including some conducted in Korea, reported that older individuals are more likely to succeed after making a quitting attempt [8,9,10,11,12]. In contrast, one survey showed that older cigarette smokers are less likely than younger adults to be interested in quitting smoking, making quitting attempts, and achieving smoking cessation [13]. Many older smokers are unwilling to quit because of the mistaken belief that it is too late to quit as damage has already occurred, that they are genetically less susceptible to smoking-attributable harm, and/or that they will not benefit greatly from quitting [14]. Smokers who start smoking later in life tend to be more likely to quit [14,15,16,17]. Other studies showed that young smokers are more likely to make a quitting attempt [18], while older individuals are more likely to succeed if they make such an attempt [9,10,11].

It is not yet clear whether it is easier for young or old individuals to quit smoking, but quitting success rates likely vary by age. Therefore, we hypothesised that factors related to successful smoking cessation may also vary with age. Accordingly, this study examined the factors affecting smoking cessation by age among Korean adult male smokers using nationally representative data. Self-reports of tobacco smoking by Korean women are largely unreliable, probably owing to the social prejudice against female Korean smokers [19,20]. Therefore, we decided to focus on the smoking cessation factors by age among Korean men alone.

## 2. Materials and Methods

### 2.1. Study Design, Data Sources, and Inclusion/Exclusion Criteria

This study was a secondary analysis of data from the Korea Health and Nutrition Survey (KNHANES), which was conducted over 11 years (2007 to 2018, excluding the year 2013). The KNHANES is an ongoing nationwide representative cross-sectional survey that examines the overall health, lifestyle behaviors, and dietary habits of the South Korean general population [7]. To minimise sampling bias, KNHANES adopted a complex stratified multistage probability-cluster sampling design based on geographic area, age, and gender. It comprises physical and mental health-related questionnaires, laboratory tests, and nutrition surveys. The data were downloaded after the designated registration process was completed for access to the official KNHANES website (http://knhanes.cdc.go.kr/). The data from the 4th to 7th (2007–2018) KNHANES surveys were analysed in this study. The 2013 data were not used as they did not include detailed information on smoking cessation.

**Figure 1:**
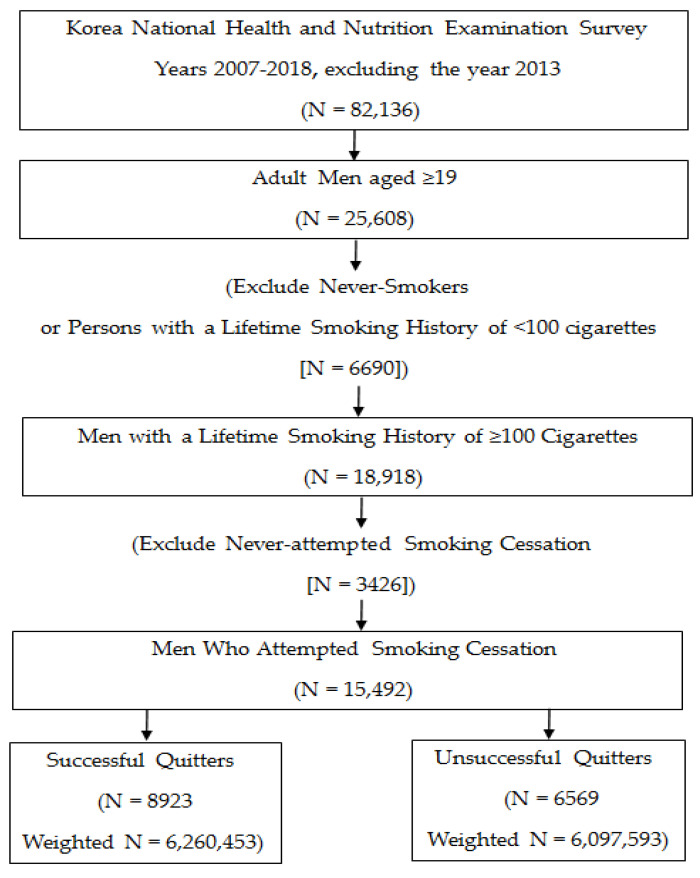
Flow diagram showing the inclusion and exclusion of study participants.

A total of 82,136 individuals participated in the survey. The study involved men aged over 19 years who had smoked more than 100 cigarettes in their lifetime and had tried to quit smoking. We excluded those who had never smoked, had smoked less than 100 cigarettes, or had never attempted to quit smoking in their lifetime. We included 15,492 men in the study, 8923 (57.6%) of whom had successfully quit smoking and 6569 (42.4%) of whom had failed to quit smoking (see Figure 1).

### 2.2. Ethical Considerations

The institutional review board (IRB) of the Korea Centers for Disease Control and Prevention (KCDC) reviewed and approved the KNHANES survey. The IRB approval numbers were 2007-02CON-04-P, 2008-04EXP-01-C, 2009-01CON-03-2C, 2010-02CON-21-C, 2011-02CON-06-C, 2012-01EXP-01-2C, 2013-12EXP-03-5C, and 2018-01-03-P-A. Written informed consent was obtained from each study participant before the survey (KCDC, 2019). The present study used only existing de-identified data.

### 2.3. Measurements and Definitions of Major Clinical and General Characteristics

Successful quitters were defined as those who reported a smoking history of 100 or more cigarettes in their lifetime and who were not current smokers at the time of the survey. Unsuccessful quitters were defined as those who reported a smoking history of 100 or more cigarettes in their lifetime and were current smokers at the time of the survey despite past attempts to quit [21,22]. Study participants were classified into the following three age groups: 19–39 years (young adult), 40–64 years (middle-aged), and ≥65 years (older adult) [23].

Various sociodemographic characteristics and health behaviors were surveyed using self-report questionnaires. A detailed smoking-related history was taken, including lifetime smoking amount (pack-years), second-hand smoking exposure, and smoking cessation methods. Participants were able to select more than one smoking cessation modality if indicated.

We also examined several variables associated with smoking cessation. Trauma history was defined as having a medical history of at least one accident or intoxication requiring hospitalisation and/or emergency room treatment during the past year. Heavy drinkers were defined as those consuming seven or more glasses of alcohol (regardless of alcohol type) per occasion, at least twice per week. Exercising at a vigorous intensity for 20 or more minutes per session, at least three times a week, was deemed as regular exercise. Moderate exercise was defined as exercise at a moderate intensity for 30 or more minutes per session, at least five times a week; performing vigorous aerobic exercise for 1.25 h or more a week; or performing a moderate level of exercise for 2.5 h or more a week. Perceived health status was defined as the perceived level of overall health (i.e., very good/good, fair, or poor/very poor). Perceived psychological stress was defined as moderate-to-severe daily stress. Individuals with a history of angina, myocardial infarction, or stroke were classified as having cardiovascular disease (CVD). Cancer was defined as having a medical history of any type of cancer.

### 2.4. Statistical Analysis

All data have been presented as mean ± standard error (SE) for continuous variables or as proportions (SE) for categorical variables. T-tests and chi-square tests were performed to assess the differences between groups for continuous and categorical variables, respectively. The prevalence of successful and unsuccessful quitters according to age group was presented as a proportion (SE). Multiple logistic regression analyses were performed to investigate the association between smoking cessation and various variables by age group. The results were reported using adjusted odds ratios (ORs) and 95% confidence intervals (CIs). Interactions between *p*-values by age group were evaluated using the Wald test and logistic regression.

As data from the KNHANES were derived using stratified and multistage clustered probability sampling methods to represent the entire Korean population, population weightings were also applied in the analyses [24]. The PROC SURVEY procedure was used to apply stratification, primary sampling units, and population weights. Also, the KNHANES provides time series weights to evaluate the yearly trend in smoking cessation data. We considered these data sufficient and, hence, did not use models with auto-correlated or conditional heteroscedastic errors.

Data were analyzed using SAS version 9.4 (SAS Institute, Inc., Cary, NC, USA). A *p*-value < 0.05 was considered statistically significant.

## 3. Results

### 3.1. Prevalence of Successful vs. Unsuccessful Quitters

Table 1 shows the prevalence rates of successful and unsuccessful quitters over 11 years. Of the 3523 individuals aged 19–39 years, 31.5% succeeded in quitting, while 54.7% of the 7390 individuals aged 40–64 years and 78.6% of the 4589 individuals aged ≥65 years succeeded in quitting. Evidently, the prevalence rates of smoking cessation in all age groups have increased over time since 2007. Failure-to-quit rates were higher than success rates only in the 19–39 years age group.

### 3.2. Sociodemographic Characteristics and Smoking History of Study Participants

Table 2 displays the sociodemographic characteristics and smoking history of study participants according to smoking cessation success or failure by age group. Successful quitters were more likely to be married in all age groups. The number of pack-years was significantly higher in unsuccessful quitters belonging to the 19–39 years and 40–64 years age groups. Second-hand smoking exposure at home was more common in unsuccessful quitters in all age groups. As for the smoking cessation method, successful smokers used willpower more often, while failed smokers chose nicotine replacement therapy (NRT), education, and consulting a smokers’ quit line more frequently, across all age groups.

### 3.3. Clinical Characteristics, Health Behaviours, and Perceived Health Status of Study Participants

Table 3 shows the differences in clinical characteristics, health behaviors, and perceived health status between successful and unsuccessful quitters by age group. Some variables, such as blood pressure (BP), body mass index (BMI), and waist circumference, varied depending on age group and success (or failure) in smoking cessation. However, heavy drinking, skipping breakfast or lunch, and perceived psychological stress were more frequently observed in those who failed to quit smoking in all age groups. Further, regular exercise and better perceived health status were more common in successful quitters in all age groups.

### 3.4. Factors Associated with Smoking Cessation According to Age Group

**Figure 2:**
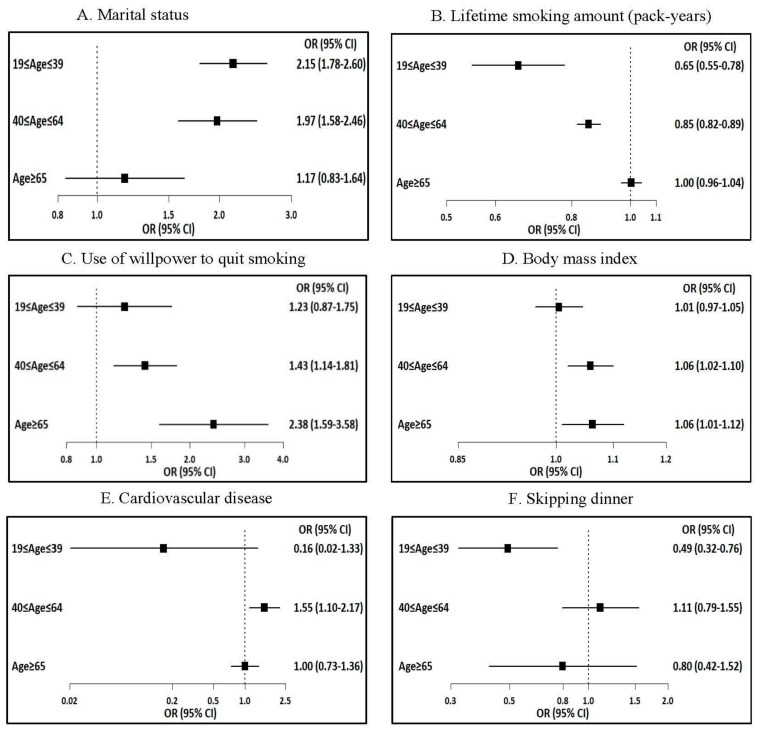
Factors associated with smoking cessation by age group.

Table 4 and Figure 2 display the various factors associated with smoking cessation according to age group. With reference to successful smoking cessation, after controlling for all variables, factors that differed significantly by age included marital status; education level; occupation; lifetime smoking amount; use of willpower, prescribed oral medicine, or counselling services to quit smoking; diastolic BP (DBP); BMI; CVD; perceived health status; and skipping dinner. Being married was associated with successful quitting only in the 19–39 years (OR 2.15, 95% CI 1.78–2.60) and 40–64 years (OR 1.97, 95% CI 1.58–2.46) age groups. Lifetime smoking was negatively associated with successful cessation in the 19–39 years (OR 0.96, 95% CI 0.94–0.98) and 40–64 years age groups (OR 0.98, 95% CI 0.98–0.99).

Willpower was related to successful cessation in those aged 40–64 years (OR 1.43, 95% CI 1.14–1.81) and ≥65 years (OR 2.38, 95% CI 1.59–3.58). A higher DBP was associated with successful cessation in those aged ≥65 years (OR 1.16, 95% CI 1.02–1.31), and a higher BMI was related to successful quitting in the 40–64 years (OR 1.06, 95% CI 1.02–1.10) and ≥65 years (OR 1.06, 95% CI, 1.01–1.12) age groups. Skipping dinner was negatively associated with successful smoking cessation only in those aged 19–39 years (OR 0.49, 95% CI 0.32–0.76). Of note, using NRT to quit and skipping breakfast or lunch were negatively associated with successful smoking cessation in all age groups.

## 4. Discussion

Although several adult cigarette smokers want to quit and have attempted to do so in the past year, fewer than 1 in 10 smokers succeed in smoking cessation each year [25,26,27]. Considering how difficult it is to quit smoking, identifying the factors involved is the first step in helping individuals to do so. Several studies have been conducted in this regard, but our study examined the factors related to smoking cessation by age among Korean adult male cigarette smokers using national survey data. We performed this study because previous research, including our own, suggested that age affects the success rate of smoking cessation [8,9,10,11,12,13,14,15,16,17].

The findings of this study can be summarised as follows. First, marriage was related to successful smoking cessation in the 19–39 years and 40–64 years age groups, whereas this was not found in men aged ≥65 years. The effect of marriage on smoking cessation remains controversial. Living alone seems to negatively impact smoking, and both men and women are reportedly more likely to quit smoking when their partners quit [28,29]. However, a meta-analysis showed that interventions that enhance partner support have no impact on increasing long-term abstinence from smoking [30]. Based on the present findings, we speculate that the age difference of the study participants could explain the variability in the findings of previous studies.

Second, the present study also found that the use of willpower—the cold turkey method—as an attempt to quit was associated with successful quitting in those aged 40–64 years and ≥65 years, but not for those in the 19–39 years age group. The smoking cessation modes included in this study were willpower, NRT, pharmacotherapy, education, counselling, and consulting a smokers’ quit line, all of which have been identified as effective smoking cessation measures in previous studies [31,32,33]. However, measures other than willpower did not help smoking cessation efforts in our study participants. Intriguingly, using NRT to quit was negatively associated with successful smoking cessation in all age groups. Of note, most study participants chose to quit smoking using willpower. This pattern of smoking cessation in our study participants may be attributable to their Korean ethnicity with its unique cultural characteristics, because masculinity is important to men in the Korean culture.

Third, we also observed that a greater amount of lifetime smoking was related to unsuccessful smoking cessation in those aged 19–39 years and 40–64 years. Smoking amount is a well-accepted factor in predicting quitting failure, which is explained by nicotine dependence. Smokers with a higher level of nicotine dependence are less likely to attempt to quit and they find it more difficult to quit [18,34,35,36,37,38]. However, the smoking amount did not affect smoking cessation in individuals aged ≥65 years in this study. As mentioned above, the use of willpower was the most successful mode of quitting in this age group. It seems that the degree of nicotine dependence may not significantly affect quitting success when older adult smokers use their willpower to quit smoking.

Fourth, higher DBP and presence of CVD were associated with successful quitting in those aged 40–64 years and ≥65 years, respectively. Having a higher BMI was related to success in quitting in the 40–64 years and ≥65 years groups, but not in the 19–39 years group. It has been reported that adult smokers with chronic medical comorbidities often try to quit and they use evidence-based tobacco-cessation treatment more often than smokers without comorbidities. However, their quit attempts are less likely to succeed [39]. In contrast, our findings suggest that comorbidities may contribute to smoking cessation in middle-aged and older adult men.

Next, the perception that one’s health is good or very good was associated with successful smoking cessation only in the 19–39 years and ≥65 years groups. Good perceived health status is most likely the result of smoking cessation, as reported in previous studies [40,41]. This was not obvious in the middle-aged group, and the reason for this anomaly is unclear. Possibly, individuals in this group thought that smoking reduced stress and anxiety.

Lastly, skipping breakfast or lunch was negatively associated with smoking cessation in all age groups, but skipping dinner was negatively associated with quitting only in the 19–39 years group. As skipping meals and smoking are both unhealthy behaviors, young adults may tend to engage in unhealthy behaviors more often than middle-aged and older adults. In general, unhealthy behaviors facilitate other unhealthy behaviors. Notably, skipping meals and smoking often share a common motivation, which is weight loss, because smoking is sometimes used to lose weight [6,42]. Studies have shown that nicotine suppresses appetite by increasing adrenaline and reducing insulin [43]. Although we lack detailed information to characterize the relationship between skipping meals and smoking continuance, weight loss may have been a motivator for persistent smoking among young adults in our study.

Overall, we observed that the 40–64 years and ≥65 years groups had a few common factors associated with smoking cessation, including higher BMI and the use of willpower to quit. In addition, these age groups had several comorbidities related to successful quitting, such as presence of CVD and high DBP. Therefore, we can cautiously speculate that these comorbidities may have been a stronger motivation for these individuals to quit smoking using willpower. Further, the 19–39 years group was the only group that exhibited a negative effect of skipping any meal on smoking cessation, indicating that having a healthy lifestyle is a critical component of young adults’ smoking cessation.

A few studies have examined the factors related to smoking cessation by age group; however, their study designs are completely different from ours. For instance, behavioral factors were the most important factors in young adults under the age of 20 years in one meta-analysis [44]. Further, another study found that for young adults between the ages of 18 and 24 years, the social environment played a critical role in the prevention of smoking recurrence [45]. In addition, one study showed that a lower nicotine dependency level increased the success of cessation in an older adult group, whereas an increase in age, the male gender, a low nicotine dependency level, and the use of medication increased the quitting success in an adult group [46]. These studies targeted somewhat limited age groups, whereas our study considered all age groups except the adolescent age group. Compared to these earlier studies, our study examined a more diverse set of variables, including the sociodemographic characteristics, detailed smoking history, clinical characteristics (including laboratory findings, comorbidities, and anthropometric characteristics), health behaviors, and health perceptions of the study participants. Most importantly, our study directly examined differences in the factors associated with smoking cessation according to age. Therefore, it is difficult to directly compare our study with others studies.

In sum, the factors related to smoking cession in this study are in line with previous research. However, we observed that factors influencing smoking cessation varied with age.

There are a few limitations to this study. The studies show that previous attempts to quit smoking and the durations of such quitting attempts can predict the success of another quitting attempt and that smokers who have recently attempted to quit smoking are more likely attempt it again. Further, smokers who quit for a longer duration in a previous attempt are more likely to successfully quit smoking [18]. Therefore, a smoker’s quitting attempt history plays a very important role in smoking cessation. However, we could not obtain detailed information on individuals’ previous quitting attempts.

In addition, the simple definitions of a successful quitter and an unsuccessful quitter used in this study might cause the misrepresentation of individuals’ smoking history.

## 5. Conclusions

This study explored the factors affecting smoking cessation by age group among Korean adult male smokers using national survey data. We observed that the different factors associated with the success or failure of smoking cessation varied by age. These factors should be considered when developing smoking cessation policies and programs.

## Figures and Tables

**Table 1:** Prevalence of successful and unsuccessful quitters by age group over the survey period (N = 15,492).

Year	Age Group											
	19–39 Years (N = 3523)			40–64 Years (N = 7380)			≥65 Years (N = 4589)			All Age Groups (N = 15,492)		
	Successful Quitters % (SE) (N = 1157) ^1^	Unsuccessful Quitters % (SE) (N = 2366) ^2^	*p*	Successful Quitters % (SE) (N = 4188) ^3^	Unsuccessful Quitters % (SE) (N = 3192) ^4^	*p*	Successful Quitters % (SE) (N = 3578) ^5^	Unsuccessful Quitters % (SE) (N = 1011) ^6^	*p*	Successful Quitters % (SE) (N = 8923) ^7^	Unsuccessful Quitters % (SE) (N = 6569) ^8^	*p*
			0.012			0.001			0.022			<0.001
2007	32.1 (3.25)	67.9 (3.25)		57.8 (3.06)	42.2 (3.06)		75.3 (4.34)	24.7 (4.34)		49.3 (2.05)	50.7 (2.05)	
2008	30.0 (2.40)	70.0 (2.40)		56.2 (2.16)	43.8 (2.16)		72.1 (3.06)	27.9 (3.06)		48.7 (1.45)	51.3 (1.45)	
2009	27.7 (2.09)	72.3 (2.09)		49.9 (1.94)	50.1 (1.94)		79.6 (2.01)	20.4 (2.01)		46.3 (1.32)	53.7 (1.32)	
2010	28.2 (2.62)	71.8 (2.62)		50.1 (2.27)	49.9 (2.27)		74.9 (2.37)	25.1 (2.37)		46.6 (1.72)	53.4 (1.72)	
2011	26.6 (2.61)	73.4 (2.61)		53.3 (2.12)	46.7 (2.12)		73.9 (2.57)	26.1 (2.57)		47.6 (1.69)	52.4 (1.69)	
2012	31.2 (3.04)	68.8 (3.04)		54.7 (2.24)	45.3 (2.24)		76.5 (2.26)	23.5 (2.26)		51.3 (1.59)	48.7 (1.59)	
2014	30.2 (3.16)	69.8 (3.16)		47.0 (2.67)	53.0 (2.67)		79.1 (2.27)	20.9 (2.27)		48.9 (1.96)	51.1 (1.96)	
2015	32.8 (2.98)	67.2 (2.98)		57.2 (2.15)	42.8 (2.15)		80.7 (2.30)	19.3 (2.30)		55.0 (1.47)	45.0 (1.47)	
2016	32.4 (3.00)	67.6 (3.00)		58.4 (2.55)	41.6 (2.55)		79.8 (2.31)	20.2 (2.31)		55.2 (1.77)	44.8 (1.77)	
2017	38.8 (3.46)	61.2 (3.46)		58.4 (2.53)	41.6 (2.53)		80.3 (2.13)	19.7 (2.13)		57.7 (1.66)	42.3 (1.66)	
2018	41.6 (3.06)	58.4 (3.06)		59.1 (1.98)	40.9 (1.98)		84.3 (2.14)	15.7 (2.14)		59.6 (1.50)	40.4 (1.50)	

Data have been presented as weighted percentage (standard error, SE). The *p*-value was determined using the Rao–Scott chi-square test. Data were not collected in 2013. ^1^ Weighted n = 1,304,511 (31.5%); ^2^ Weighted n = 2,832,741 (68.5%); ^3^ Weighted n = 3,442,040 (54.7%); ^4^ Weighted n = 2,851,877 (45.3%); ^5^ Weighted n = 1,513,902 (78.6%); ^6^ Weighted n = 412,976 (21.4%); ^7^ Weighted n = 6,260,453 (50.7%); ^8^ Weighted n = 6,097,593 (49.3%).

**Table 2:** Sociodemographic characteristics and smoking history of study participants by age group (N = 15,492).

Variables	Age 19–39 Years (N = 3523)			Age 40–64 Years (N = 7380)			Age ≥65 Years (N = 4589)		
	Successful Quitters (N = 1157)	Unsuccessful Quitters (N = 2366)	*p*	Successful Quitters (N = 4188)	Unsuccessful Quitters (N = 3192)	*p*	Successful Quitters (N = 3578)	Unsuccessful Quitters (N = 1011)	*p*
Married (%)	57.6 (1.69)	44.9 (1.23)	<0.001	91.6 (0.52)	83.9 (0.79)	<0.001	89.3 (0.64)	86.4 (1.33)	0.033
Education level (%)			<0.001			0.009			<0.001
≤High school	40.9 (1.64)	54.6 (1.18)		61.9 (0.98)	65.3 (1.06)		84.1 (0.87)	90.1 (1.14)	
University or higher	59.1 (1.64)	45.4 (1.18)		38.1 (0.98)	34.7 (1.06)		15.9 (0.87)	9.9 (1.14)	
Occupation			<0.001			0.198			<0.001
Manager/Professionals	22.5 (1.37)	18.5 (0.89)		18.3 (0.79)	16.7 (0.79)		3.3 (0.38)	3.0 (0.75)	
Office worker	20.6 (1.25)	14.2 (0.77)		13.2 (0.63)	12.3 (0.71)		1.4 (0.24)	1.9 (0.50)	
Service workers/Sellers	11.6 (1.05)	17.9 (0.92)		13.6 (0.63)	13.1 (0.71)		2.9 (0.33)	2.9 (0.56)	
Agriculture/Fishery/Labour	26.2 (1.42)	28.9 (1.05)		42.4 (0.95)	45.6 (1.12)		28.7 (1.03)	38.4 (1.98)	
None	19.1 (1.41)	20.5 (1.08)		12.5 (0.60)	12.3 (0.67)		63.7 (1.09)	53.8 (1.99)	
Residence (% Rural)	10.1 (1.19)	12.5 (1.07)	0.064	18.0 (1.04)	22.3 (1.31)	0.001	25.2 (1.37)	29.9 (1.98)	0.008
Household income (Quartiles)			0.062			<0.001			0.001
1st (Lowest)	23.1 (1.37)	25.9 (1.06)		21.2 (0.78)	27.9 (0.97)		23.6 (0.88)	28.8 (1.73)	
2nd	24.7 (1.39)	24.9 (0.98)		25.8 (0.82)	27.0 (0.96)		23.9 (0.85)	26.1 (1.65)	
3rd	24.8 (1.38)	26.1 (1.02)		25.9 (0.81)	23.6 (0.89)		24.5 (0.86)	24.2 (1.60)	
4th (Highest)	27.3 (1.48)	23.1 (1.05)		27.1 (0.87)	21.5 (0.90)		27.9 (0.97)	20.9 (1.60)	
Lifetime smoking amount (pack year)	6.93 ± 0.22	8.67 ± 0.17	<0.001	19.26 ± 0.30	24.87 ± 0.30	<0.001	29.32 ± 0.54	30.51 ± 0.82	0.220
Second-hand smoking (%)									
Workplace (yes)	43.4 (1.72)	46.4 (1.19)	0.148	36.7 (0.89)	44.1 (1.03)	<0.001	8.7 (0.57)	13.4 (1.32)	<0.001
Home (yes)	4.6 (0.73)	11.8 (0.82)	<0.001	1.8 (0.24)	5.2 (0.44)	<0.001	2.4 (0.32)	3.8 (0.68)	0.026
Smoking cessation methods (%)									
Willpower	94.2 (0.72)	87.1 (0.76)	<0.001	92.8 (0.47)	82.3 (0.80)	<0.001	95.3 (0.44)	81.9 (1.48)	<0.001
Nicotine replacement therapy	6.0 (0.75)	16.8 (0.84)	<0.001	5.8 (0.42)	17.5 (0.79)	<0.001	3.3 (0.35)	11.4 (1.29)	<0.001
Prescribed oral medicines	1.0 (0.31)	0.9 (0.22)	0.809	1.2 (0.20)	2.0 (0.28)	0.020	0.8 (0.18)	3.0 (0.68)	<0.001
Education/Counselling	4.1 (0.59)	8.6 (0.62)	<0.001	5.4 (0.40)	12.7 (0.67)	<0.001	3.9 (0.41)	17.4 (1.42)	<0.001
Smokers’ quit line	0.4 (0.18)	0.7 (0.17)	0.296	0.8 (0.17)	1.9 (0.27)	<0.001	0.4 (0.12)	1.5 (0.44)	<0.001

Data have been presented as weighted mean ± SE or weighted percentage (SE). The *p*-value was determined using the Student’s *t*-test or Rao–Scott chi-square test. Income quartiles were adjusted for age and gender.

**Table 3:** Clinical characteristics, health behaviors, and perceived health status of study participants by age group (N = 15,492).

Variables	Age 19–39 Years (N = 3523)			Age 40–64 Years (N = 7380)			Age ≥65 Years (N = 4589)		
	Successful Quitters (N = 1157)	Unsuccessful Quitters (N = 2366)	*p*	Successful Quitters (N = 4188)	Unsuccessful Quitters (N = 3192)	*p*	Successful Quitters (N = 3578)	Unsuccessful Quitters (N = 1011)	*p*
Systolic blood pressure (mmHg)	114.96 ± 0.40	115.07 ± 0.28	0.820	121.76 ± 0.28	119.58 ± 0.32	<0.001	127.37 ± 0.36	125.87 ± 0.65	0.035
Diastolic blood pressure (mmHg)	77.37 ± 0.36	77.09 ± 0.25	0.513	81.31 ± 0.19	79.89 ± 0.23	<0.001	73.30 ± 0.22	71.49 ± 0.41	<0.001
Body mass index (kg/m^2^)	24.60 ± 0.12	24.44 ± 0.09	0.272	24.76 ± 0.05	24.21 ± 0.06	<0.001	23.57 ± 0.06	22.86 ± 0.12	<0.001
Waist circumference (cm)	84.72 ± 0.32	84.20 ± 0.24	0.185	86.67 ± 0.15	85.60 ± 0.18	<0.001	86.18 ± 0.18	84.36 ± 0.35	<0.001
Fasting blood sugar (mg/dL)	94.18 ± 0.48	93.04 ± 0.39	0.065	104.78 ± 0.43	104.47 ± 0.55	0.660	107.31 ± 0.54	106.18 ± 1.12	0.368
Total cholesterol (mg/dL)	187.16 ± 1.09	187.26 ± 0.88	0.945	194.72 ± 0.67	194.55 ± 0.74	0.865	178.90 ± 0.77	178.08 ± 1.59	0.645
Diabetes mellitus (%)	13.0 (1.08)	12.0 (0.78)	0.493	38.2 (0.86)	31.3 (0.97)	<0.001	58.4 (0.99)	53.4 (1.94)	0.020
Hypertension (%)	2.2 (0.48)	2.5 (0.36)	0.680	14.2 (0.62)	14.3 (0.68)	0.919	23.7 (0.89)	23.8 (1.72)	0.957
Cardiovascular disease (%)	0.4 (0.22)	0.4 (0.12)	0.747	4.7 (0.36)	3.3 (0.34)	0.005	16.0 (0.75)	14.5 (1.34)	0.339
Cancer (%)	0.4 (0.18)	0.2 (0.09)	0.255	3.5 (0.30)	1.6 (0.26)	<0.001	10.2 (0.60)	6.3 (0.94)	0.002
Trauma history (%)	7.9 (0.94)	11.0 (0.73)	0.012	7.4 (0.48)	8.4 (0.61)	0.162	5.5 (0.49)	9.1 (1.09)	0.001
Heavy drinker (%)	22.4 (1.37)	29.7 (1.03)	<0.001	22.2 (0.77)	31.1 (0.93)	<0.001	8.2 (0.54)	11.6 (1.26)	0.007
Regular exercise	43.8 (1.63)	39.7 (1.19)	0.039	40.9 (0.96)	32.8 (0.97)	<0.001	31.6 (1.02)	26.2 (1.75)	0.008
Skipping meals (%)									
Skipping breakfast	34.6 (1.60)	45.6 (1.23)	<0.001	14.1 (0.68)	21.6 (0.89)	<0.001	3.1 (0.37)	8.9 (1.28)	<0.001
Skipping lunch	6.9 (0.84)	11.3 (0.86)	0.007	5.1 (0.43)	6.9 (0.55)	0.009	5.1 (0.46)	10.7 (1.35)	<0.001
Skipping dinner	4.0 (0.61)	6.2 (0.57)	0.012	3.5 (0.35)	4.2 (0.43)	0.195	2.6 (0.33)	3.8 (0.75)	0.105
Perceived health status (%)			<0.001			<0.001			<0.001
Very good/Good	48.7 (1.59)	35.5 (1.13)		37.3 (0.87)	30.6 (0.95)		30.1 (0.92)	23.9 (1.58)	
Fair	41.5 (1.57)	50.4 (1.15)		48.4 (0.90)	52.2 (1.03)		43.2 (1.03)	43.0 (1.90)	
Poor/Very poor	9.9 (1.00)	14.1 (0.83)		14.3 (0.60)	17.2 (0.76)		26.7 (0.90)	33.0 (1.79)	
Perceived psychological stress (%)	28.7 (1.42)	35.0 (1.09)	0.001	21.8 (0.73)	30.3 (0.90)	<0.001	12.9 (0.68)	16.1 (1.32)	0.023

Data have been presented as weighted mean ± SE or weighted percentage (SE). The *p*-value was determined using the Student’s *t*-test or Rao–Scott chi-square test.

**Table 4:** Factors associated with smoking cessation by age group.

Variables	Age 19–39 Years	Age 40–64 Years	Age ≥65 Years	*p* for Interaction
	OR (95% CI)	OR (95% CI)	OR (95% CI)	
Marital status (Married)	2.15 (1.78–2.60)	1.97 (1.58–2.46)	1.17 (0.83–1.64)	0.007
Education level (University)	1.31 (1.08–1.69)	0.98 (0.85–1.15)	0.93 (0.65–1.33)	0.043
Occupation (Ref, None)				0.013
Manager/Professionals	0.85 (0.62–1.16)	0.75 (0.58–0.98)	0.57 (0.26–1.24)	
Office workers	0.98 (0.71–1.35)	0.76 (0.58–1.00)	0.30 (0.14–0.63)	
Service workers/Sellers	0.56 (0.40–0.78)	0.82 (0.63–1.07)	0.60 (0.36–0.99)	
Agriculture/Fishery/Labour	0.78 (0.58–1.06)	0.83 (0.66–1.02)	0.63 (0.50–0.79)	
Residence (Rural)	0.84 (0.63–1.13)	0.87 (0.73–1.03)	0.85 (0.67–1.06)	0.972
Household income (Ref, Lowest)				0.972
2nd	1.21 (0.93–1.58)	1.10 (0.91–1.33)	1.11 (0.80–1.53)	
3rd	1.09 (0.84–1.42)	1.15 (0.95–1.39)	1.16 (0.85–1.59)	
4th (Highest)	1.35 (1.02–1.78)	1.37 (1.13–1.67)	1.26 (0.93–1.72)	
Lifetime smoking amount (Pack-year)	0.96 (0.94–0.98)	0.98 (0.98–0.99)	1.00 (1.00–1.00)	<0.001
Second-hand smoking				
Workplace (Yes)	0.93 (0.76–1.13)	0.89 (0.78–1.02)	0.77 (0.54–1.10)	0.665
Home (Yes)	0.39 (0.24–0.62)	0.42 (0.28–0.62)	0.75 (0.41–1.39)	0.198
Smoking cessation modes				
Willpower	1.23 (0.87–1.75)	1.43 (1.14–1.81)	2.38 (1.59–3.58)	0.034
Nicotine replacement therapy	0.37 (0.25–0.54)	0.38 (0.30–0.48)	0.42 (0.26–0.70)	0.897
Prescribed oral medicines	1.16 (0.45–2.99)	0.80 (0.46–1.39)	0.24 (0.10–0.54)	0.017
Education or counselling	0.69 (0.46–1.04)	0.60 (0.46–0.78)	0.33 (0.22–0.50)	0.020
Smokers’ quit line	1.00 (0.22–4.55)	0.76 (0.40–1.44)	0.74 (0.21–2.64)	0.944
Systolic BP (per 10 mmHg increase)	0.95 (0.87–1.05)	1.07 (1.00–1.13)	1.05 (0.98–1.12)	0.053
Diastolic BP (per 10 mmHg increase)	0.96 (0.87–1.07)	1.06 (0.97–1.16)	1.16 (1.02–1.31)	0.044
Body mass index (kg/m^2^)	1.01 (0.97–1.05)	1.06 (1.02–1.10)	1.06 (1.01–1.12)	0.008
Waist circumference (per 10 cm increase)	0.93 (0.79–1.09)	1.09 (0.95–1.25)	1.07 (0.92–1.25)	0.052
Fasting blood sugar (per 10 mg/dL increase)	1.06 (1.00–1.13)	1.03 (0.99–1.06)	1.02 (0.97–1.06)	0.449
Total cholesterol (per 10 mg/dL increase)	0.99 (0.96–1.02)	0.99 (0.97–1.01)	1.01 (0.98–1.05)	0.526
Hypertension	0.87 (0.64–1.18)	1.23 (1.05–1.44)	1.05 (0.84–1.31)	0.071
Diabetes mellitus	0.75 (0.41–1.38)	0.91 (0.72–1.15)	0.83 (0.62–1.11)	0.724
Cardiovascular disease	0.16 (0.02–1.33)	1.55 (1.10–2.17)	1.00 (0.73–1.36)	0.027
Cancer	1.97 (0.44–8.79)	2.22 (1.42–3.48)	1.73 (1.09–2.74)	0.735
Trauma history	0.74 (0.53–1.04)	0.96 (0.75–1.23)	0.62 (0.43–0.90)	0.131
Heavy drinker	0.69 (0.56–0.86)	0.64 (0.55–0.75)	0.76 (0.52–1.10)	0.661
Exercise	1.23 (1.02–1.49)	1.34 (1.16–1.54)	1.15 (0.90–1.47)	0.539
Perceived health status (Ref, Poor/Very poor)				0.016
Very good/Good	1.62 (1.18–2.23)	1.21 (0.99–1.49)	1.46 (1.09–1.94)	
Fair	0.97 (0.71–1.33)	1.11 (0.91–1.34)	1.18 (0.91–1.52)	
Perceived psychological stress	0.79 (0.65–0.96)	0.69 (0.60–0.80)	0.81 (0.60–1.09)	0.437
Skipping meals				
Skipping breakfast	0.67 (0.56–0.81)	0.62 (0.52–0.75)	0.36 (0.21–0.62)	0.090
Skipping lunch	0.66 (0.45–0.97)	0.75 (0.57–0.99)	0.43 (0.28–0.66)	0.089
Skipping dinner	0.49 (0.32–0.76)	1.11 (0.79–1.55)	0.80 (0.42–1.52)	0.017

OR: odds ratio; CI: confidence interval; Ref: reference; Interactions between *p*-values by age group were evaluated using the Wald test and logistic regression.
